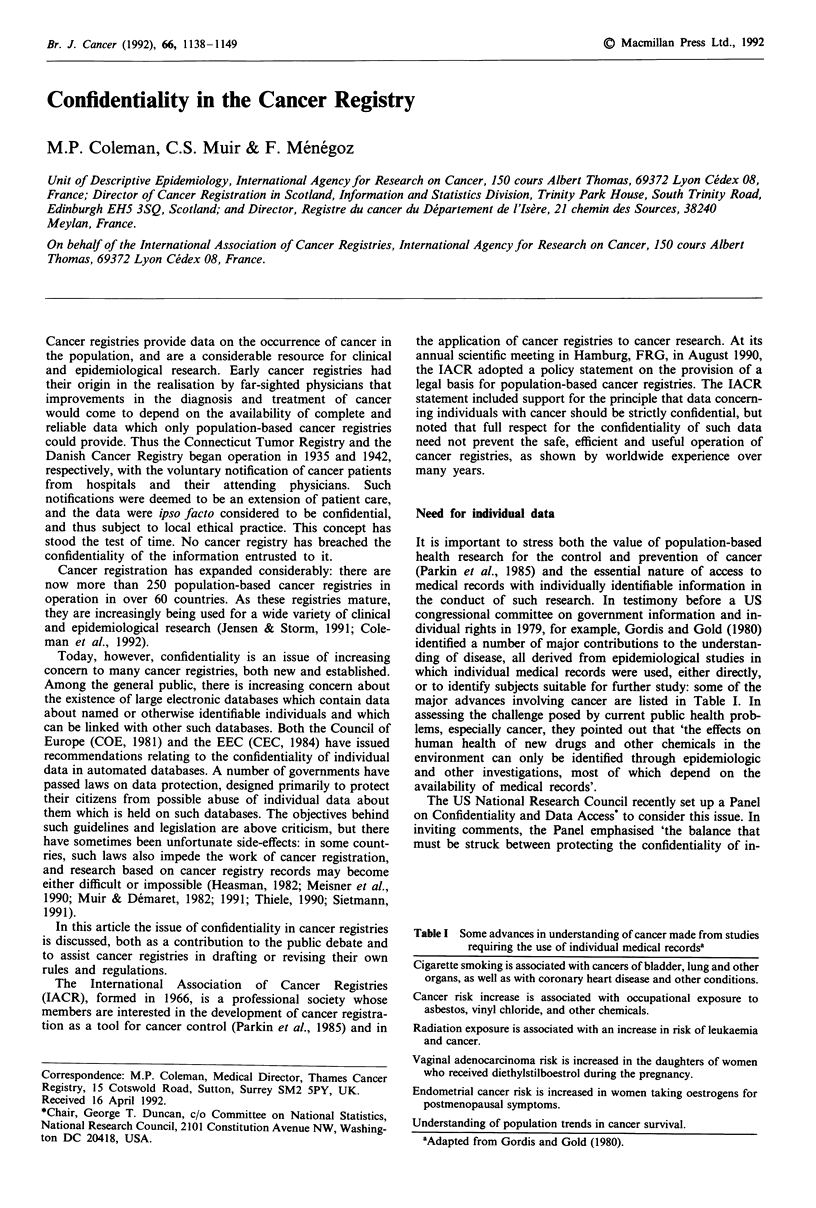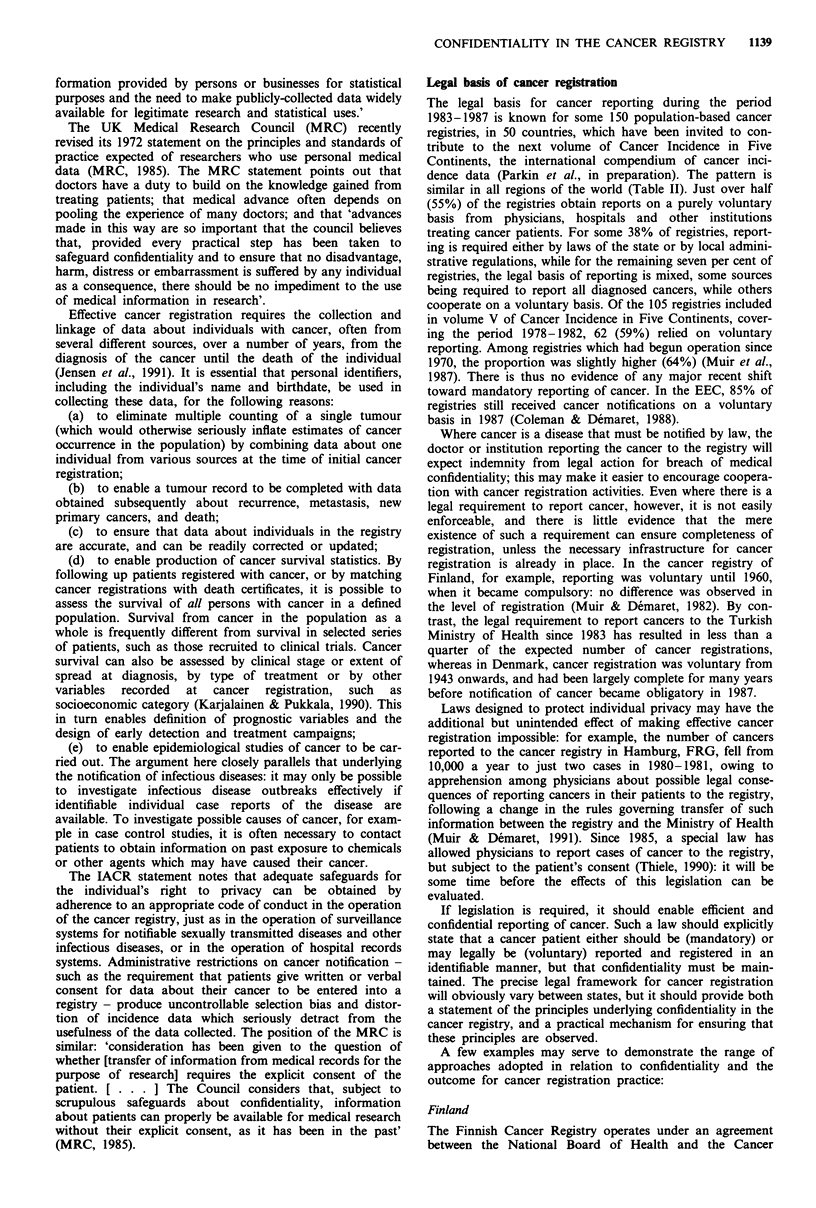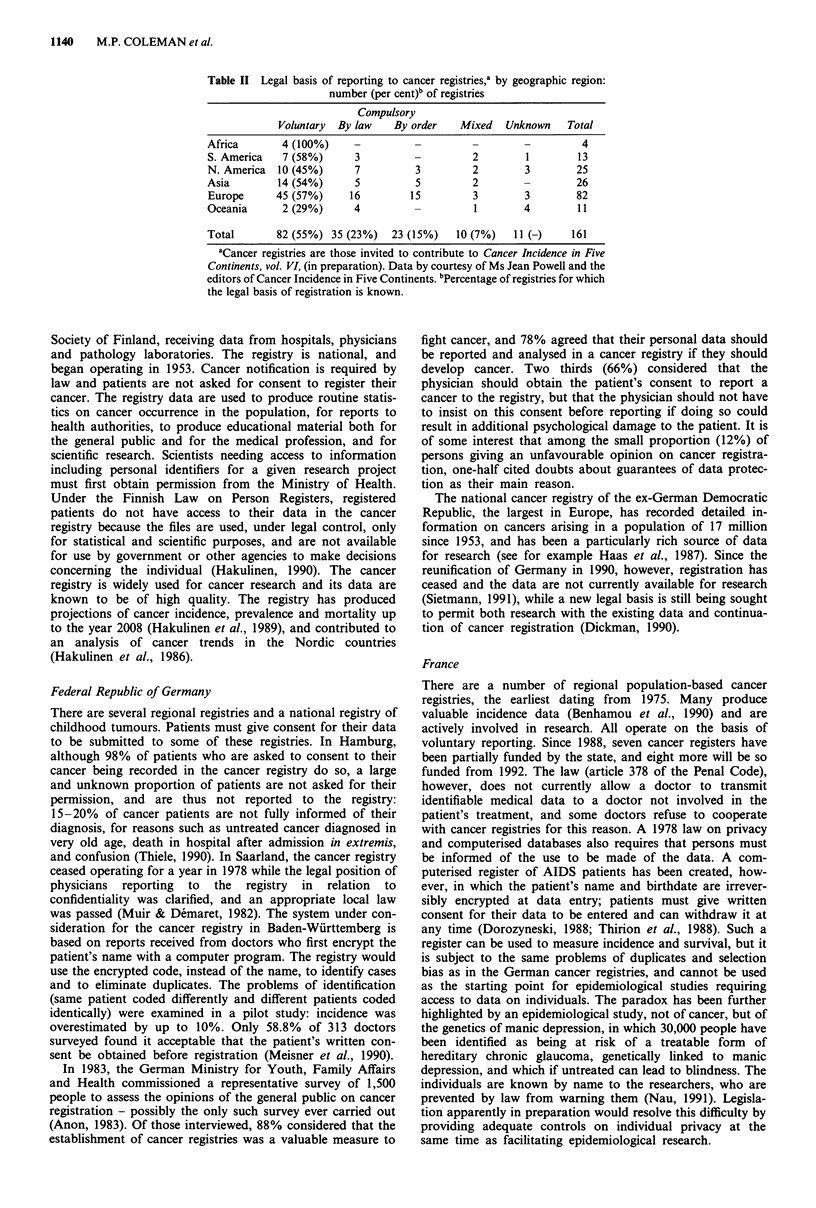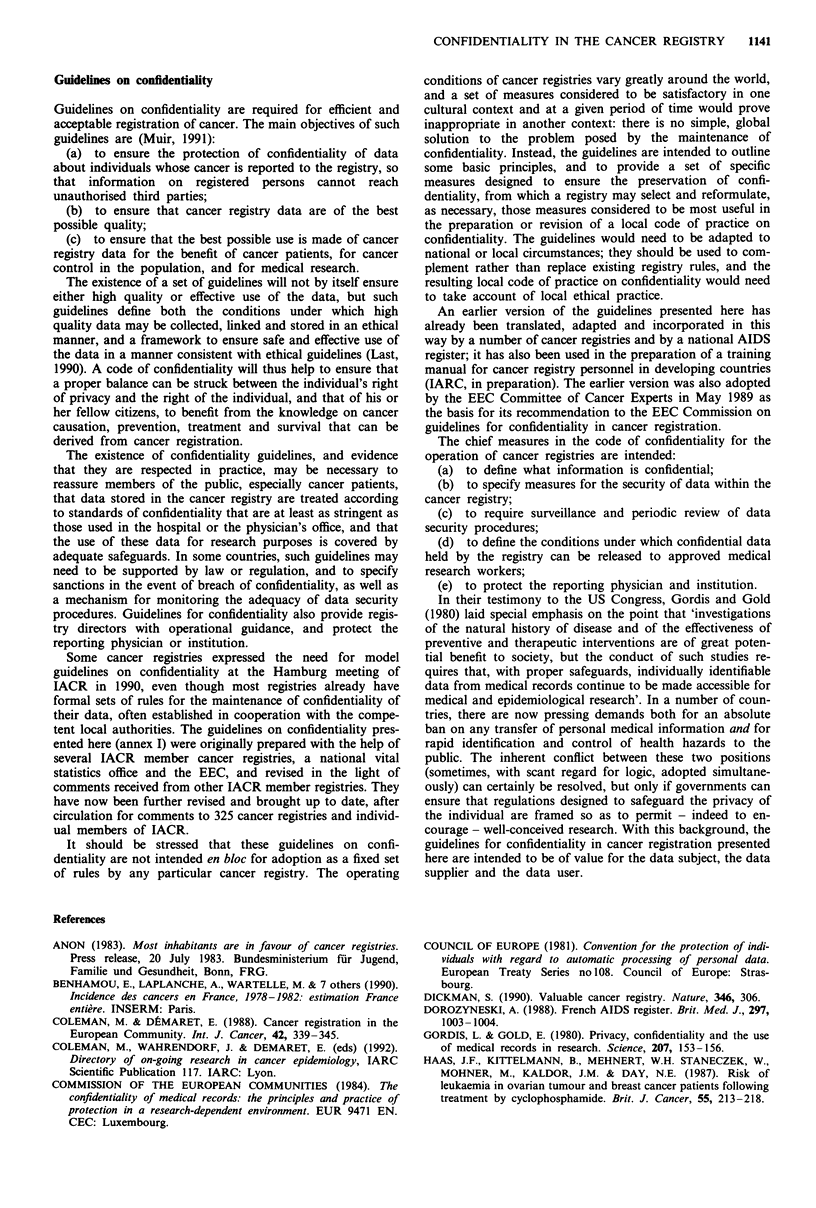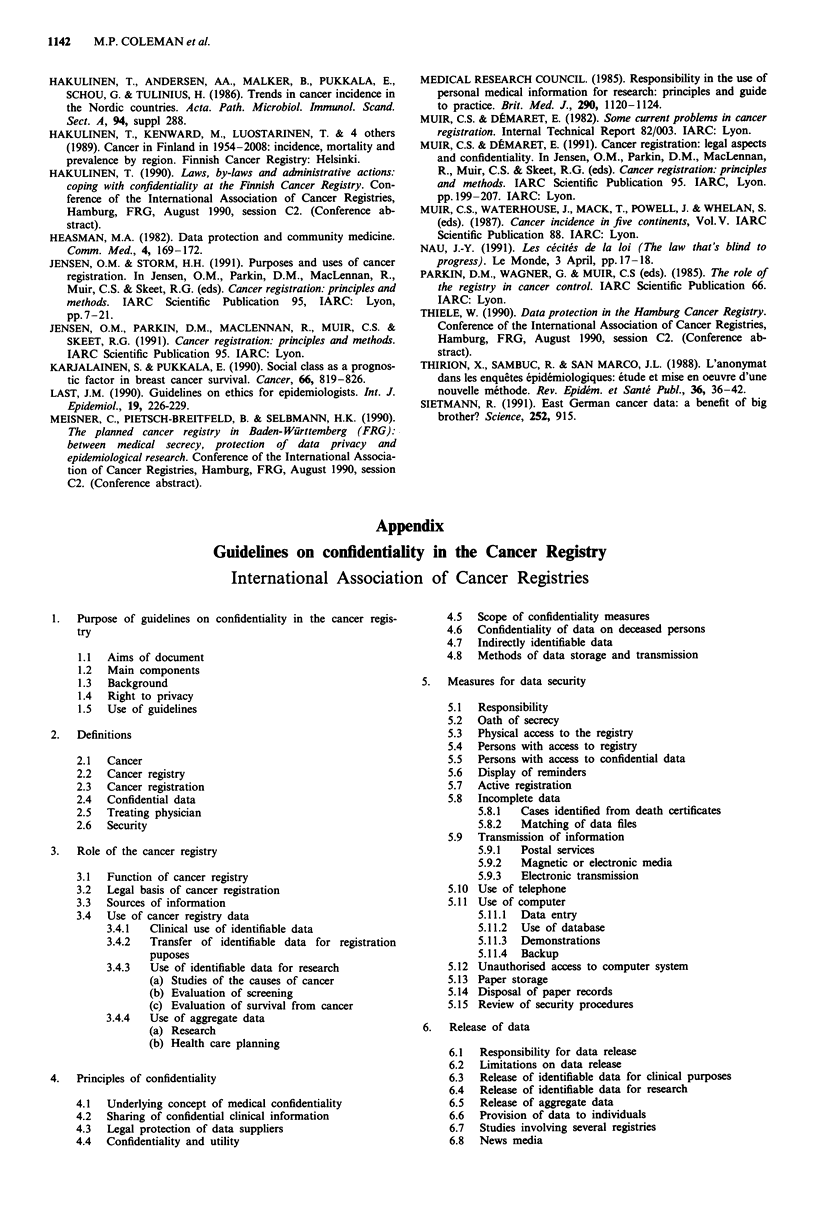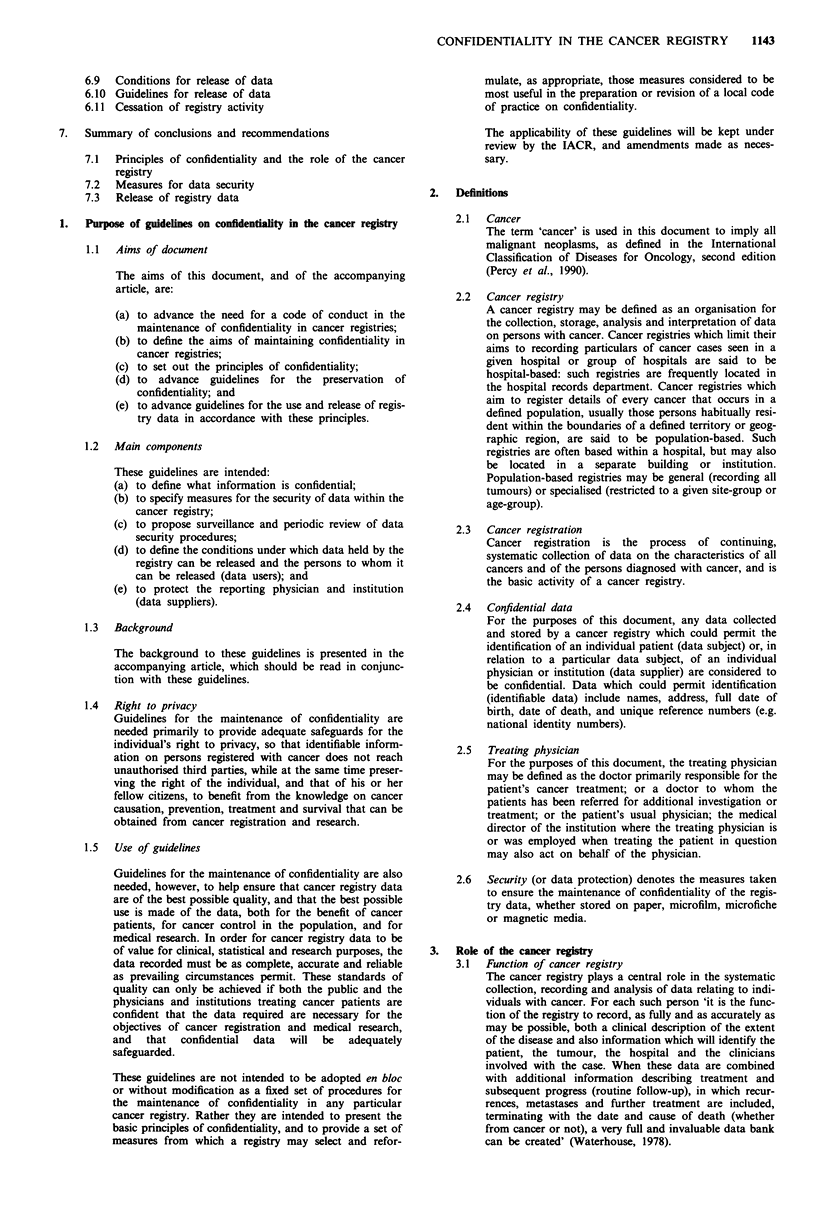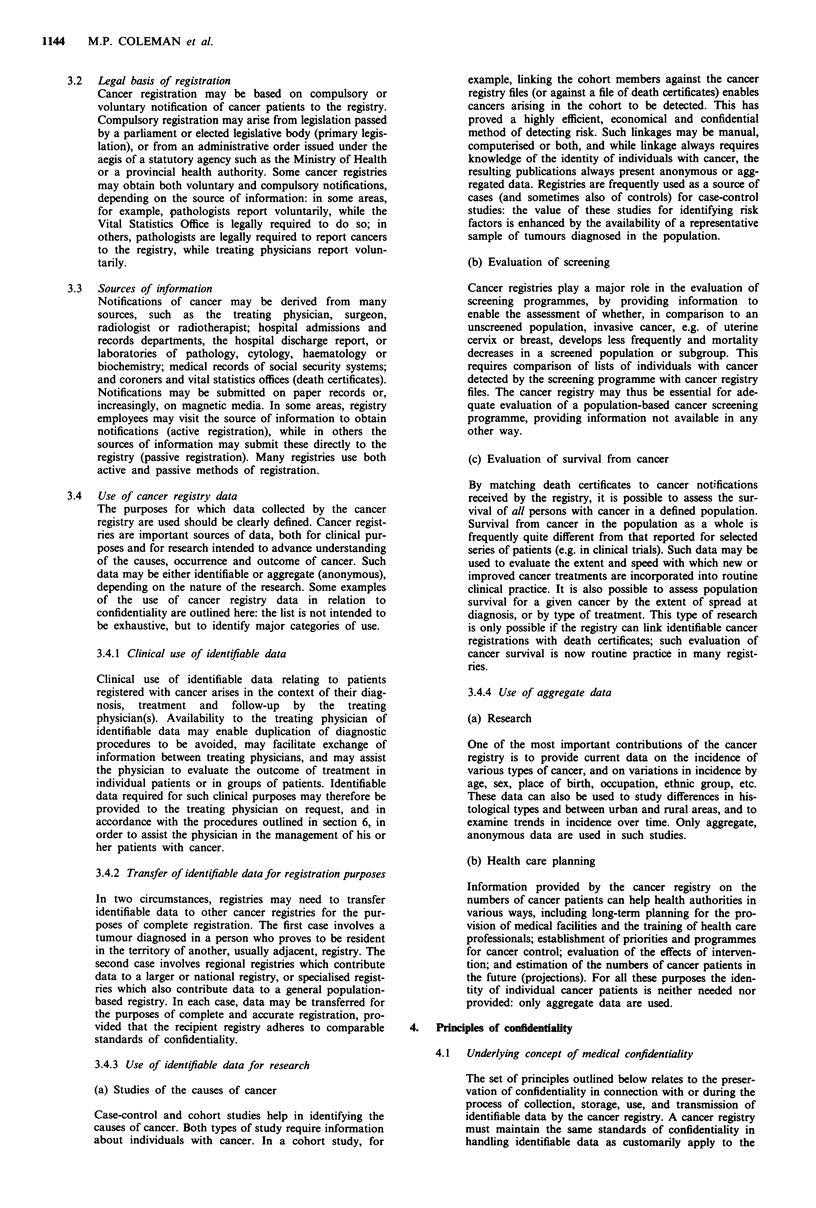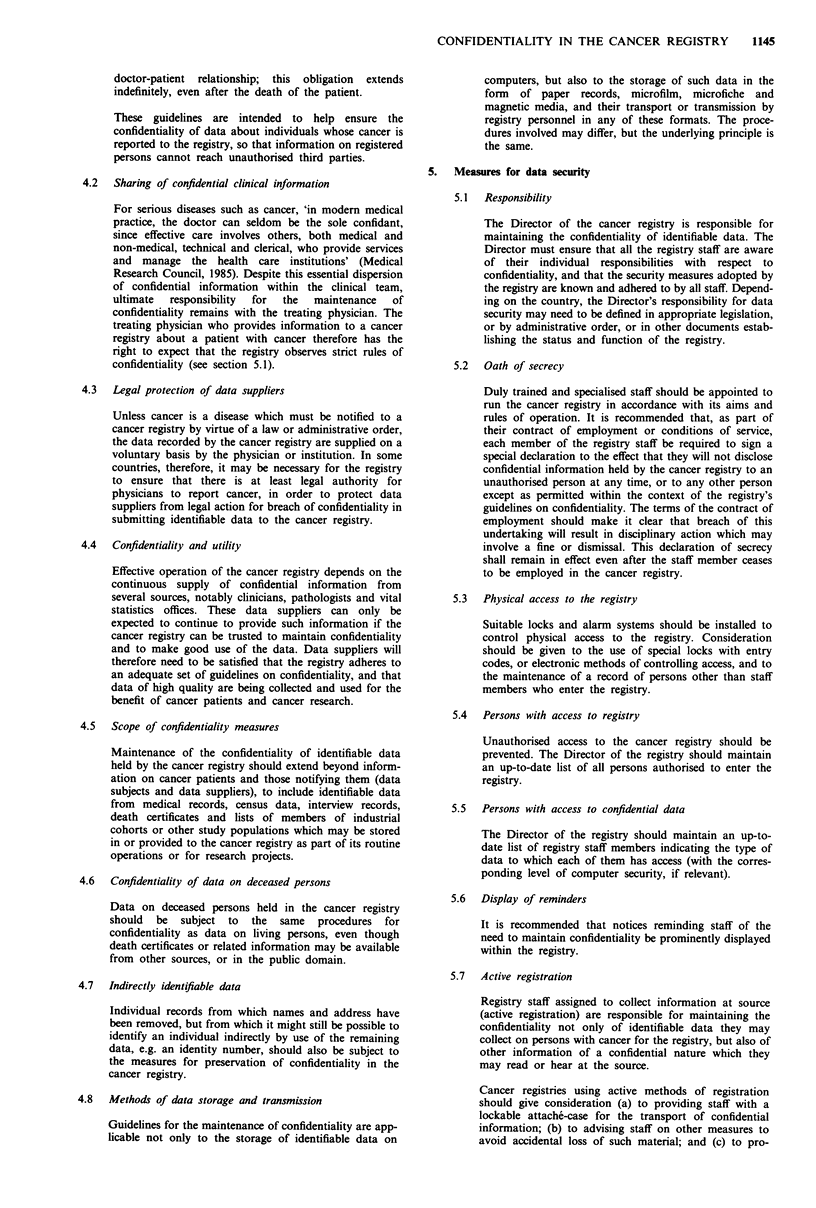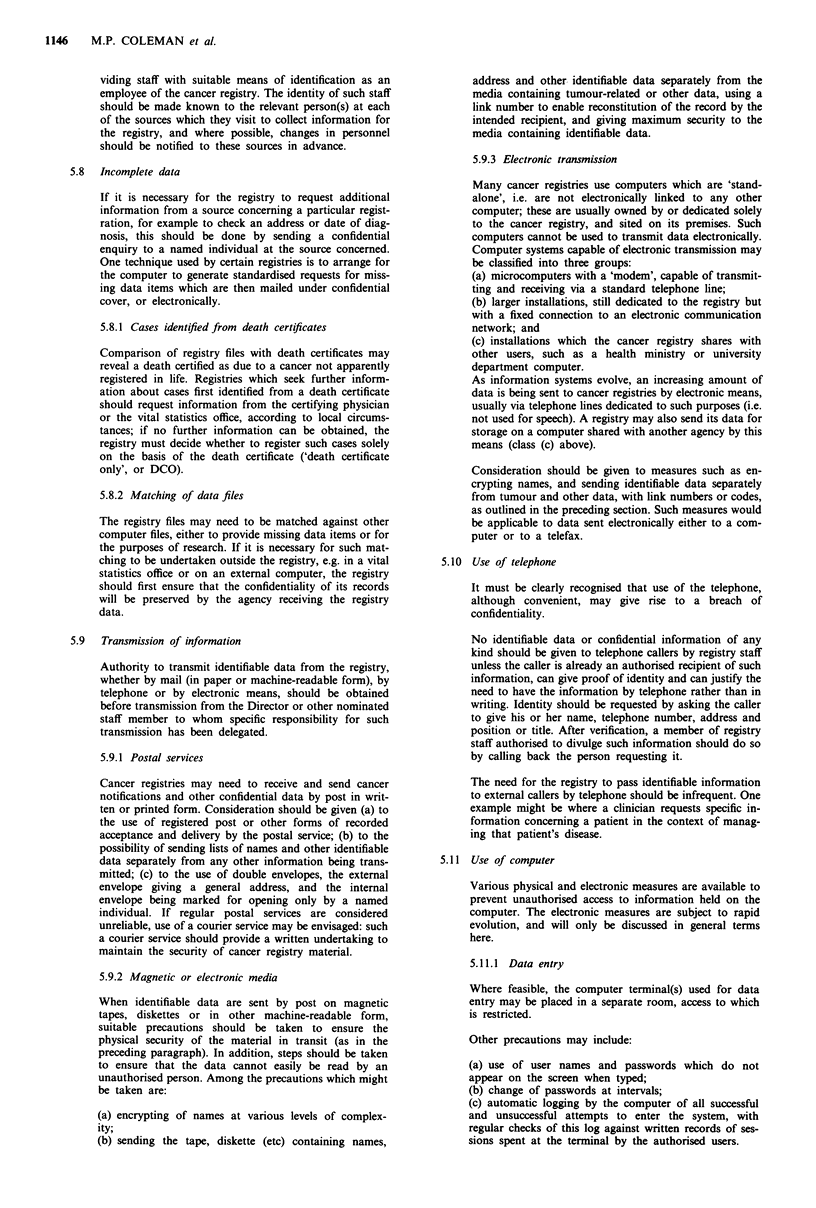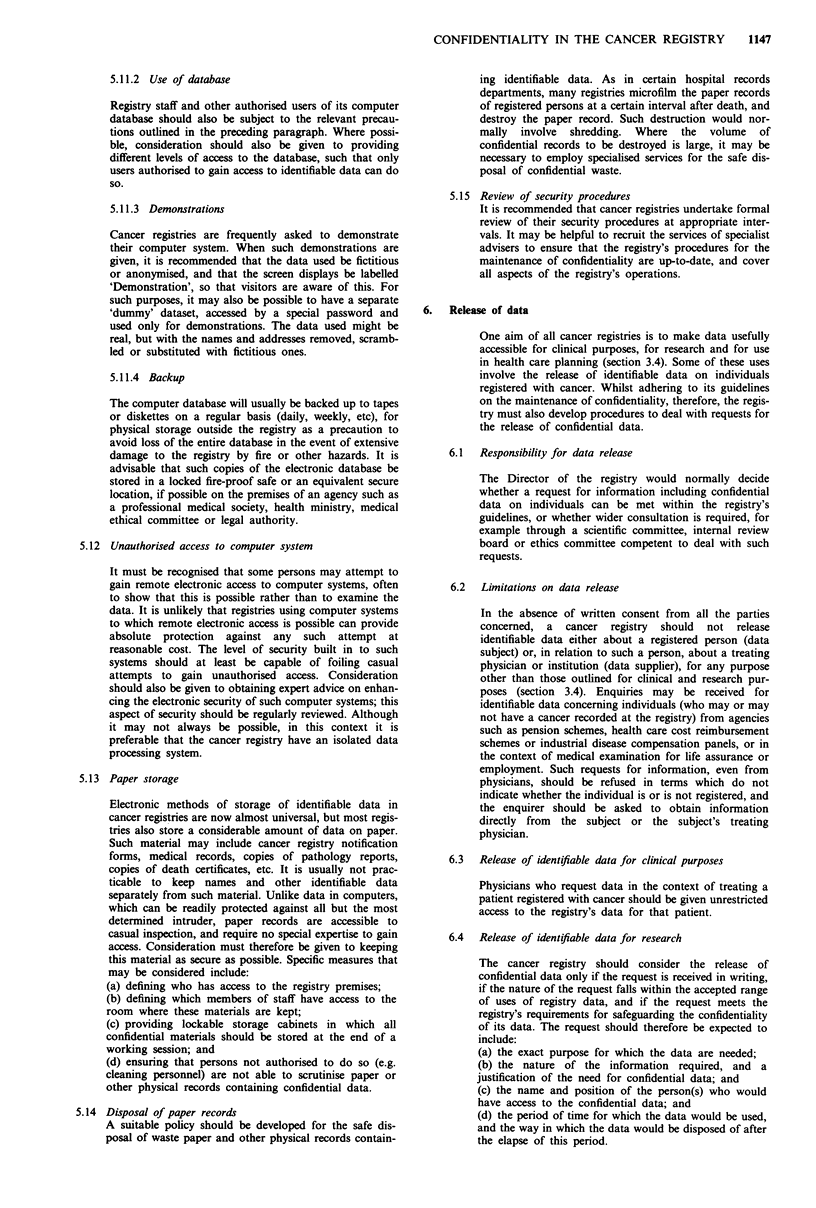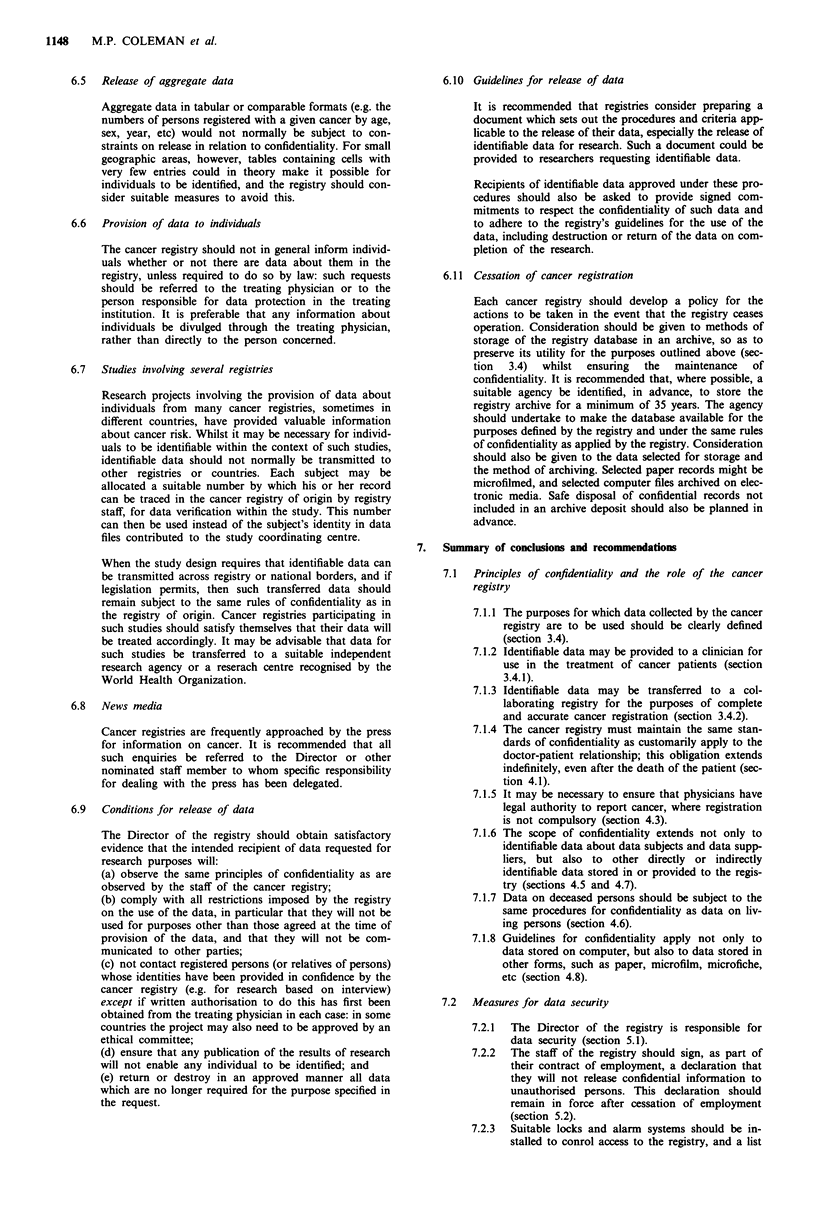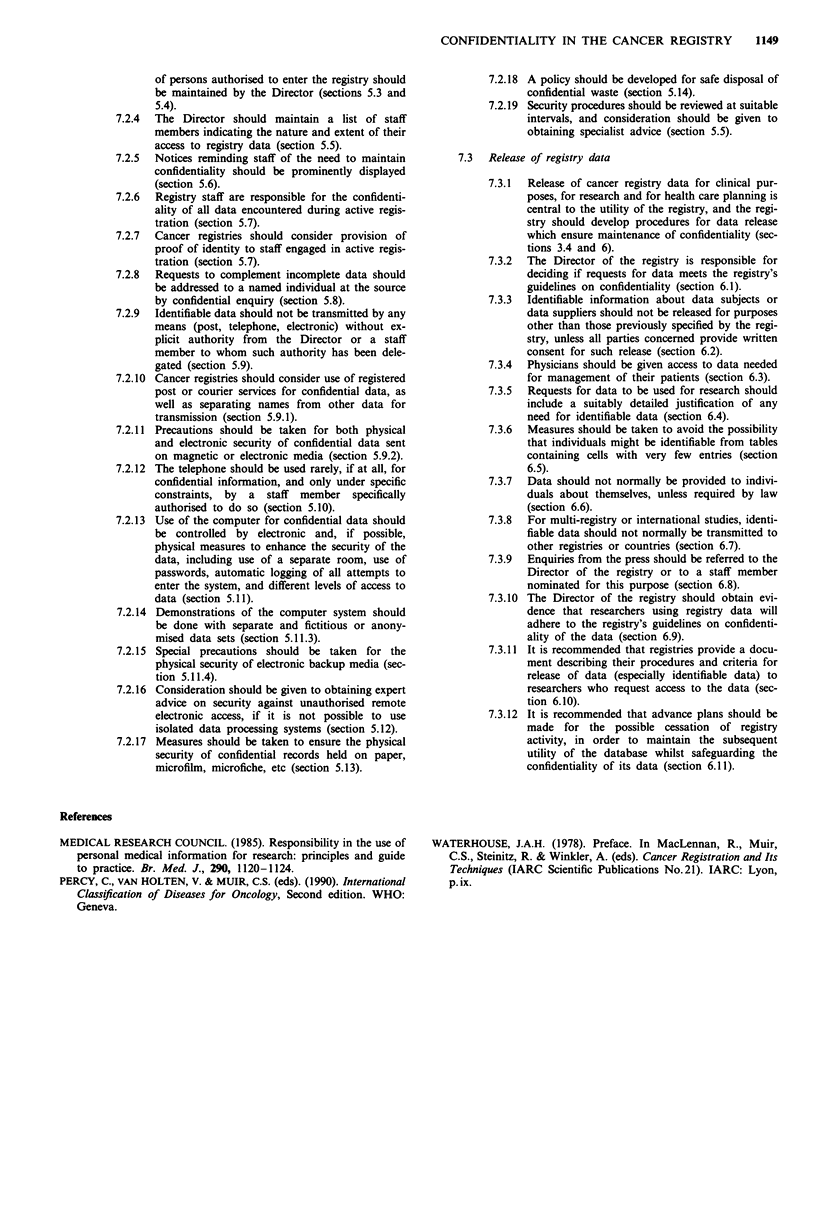# Confidentiality in the cancer registry.

**DOI:** 10.1038/bjc.1992.424

**Published:** 1992-12

**Authors:** M. P. Coleman, C. S. Muir, F. Ménégoz

**Affiliations:** Unit of Descriptive Epidemiology, International Agency for Research on Cancer, Lyon, France.


 © Macmillan Press Ltd., 1992

### Confidentiality in the Cancer Registry

M.P. Coleman, C.S. Muir & F. Ménégoz

*Unit of Descriptive Epidemiology, International Agency for Research on Cancer, 150 cours Albert Thomas, 69372 Lyon Cédex 08, France; Director of Cancer Registration in Scotland, Information and Statistics Division, Trinity Park House, South Trinity Road, Edinburgh EH5 3SQ, Scotland; and Director, Registre du cancer du Département de l'Isère, 21 chemin des Sources, 38240 Meylan, France.*

*On behalf of the International Association of Cancer Registries, International Agency for Research on Cancer, 150 cours Albert Thomas, 69372 Lyon Cédex 08, France.*

Cancer registries provide data on the occurrence of cancer in the population, and are a considerable resource for clinical and epidemiological research. Early cancer registries had their origin in the realisation by far-sighted physicians that improvements in the diagnosis and treatment of cancer would come to depend on the availability of complete and reliable data which only population-based cancer registries could provide. Thus the Connecticut Tumor Registry and the Danish Cancer Registry began operation in 1935 and 1942, respectively, with the voluntary notification of cancer patients from hospitals and their attending physicians. Such notifications were deemed to be an extension of patient care, and the data were *ipso facto* considered to be confidential, and thus subject to local ethical practice. This concept has stood the test of time. No cancer registry has breached the confidentiality of the information entrusted to it.

Cancer registration has expanded considerably: there are now more than 250 population-based cancer registries in operation in over 60 countries. As these registries mature, they are increasingly being used for a wide variety of clinical and epidemiological research (Jensen & Storm, 1991; Coleman *et al.*, 1992).

Today, however, confidentiality is an issue of increasing concern to many cancer registries, both new and established. Among the general public, there is increasing concern about the existence of large electronic databases which contain data about named or otherwise identifiable individuals and which can be linked with other such databases. Both the Council of Europe (COE, 1981) and the EEC (CEC, 1984) have issued recommendations relating to the confidentiality of individual data in automated databases. A number of governments have passed laws on data protection, designed primarily to protect their citizens from possible abuse of individual data about them which is held on such databases. The objectives behind such guidelines and legislation are above criticism, but there have sometimes been unfortunate side-effects: in some countries, such laws also impede the work of cancer registration, and research based on cancer registry records may become either difficult or impossible (Heasman, 1982; Meisner *et al.*, 1990; Muir & Démaret, 1982; 1991; Thiele, 1990; Sietmann, 1991).

In this article the issue of confidentiality in cancer registries is discussed, both as a contribution to the public debate and to assist cancer registries in drafting or revising their own rules and regulations.

The International Association of Cancer Registries (IACR), formed in 1966, is a professional society whose members are interested in the development of cancer registration as a tool for cancer control (Parkin *et al.*, 1985) and in the application of cancer registries to cancer research. At its annual scientific meeting in Hamburg, FRG, in August 1990, the IACR adopted a policy statement on the provision of a legal basis for population-based cancer registries. The IACR statement included support for the principle that data concerning individuals with cancer should be strictly confidential, but noted that full respect for the confidentiality of such data need not prevent the safe, efficient and useful operation of cancer registries, as shown by worldwide experience over many years.

### Need for individual data

It is important to stress both the value of population-based health research for the control and prevention of cancer (Parkin *et al.*, 1985) and the essential nature of access to medical records with individually identifiable information in the conduct of such research. In testimony before a US congressional committee on government information and individual rights in 1979, for example, Gordis and Gold (1980) identified a number of major contributions to the understanding of disease, all derived from epidemiological studies in which individual medical records were used, either directly, or to identify subjects suitable for further study: some of the major advances involving cancer are listed in Table I. In assessing the challenge posed by current public health problems, especially cancer, they pointed out that 'the effects on human health of new drugs and other chemicals in the environment can only be identified through epidemiologic and other investigations, most of which depend on the availability of medical records'.

The US National Research Council recently set up a Panel on Confidentiality and Data Access* to consider this issue. In inviting comments, the Panel emphasised 'the balance that must be struck between protecting the confidentiality of in-

**Table I** Some advances in understanding of cancer made from studies requiring the use of individual medical records[a]

| |
|---|
| Cigarette smoking is associated with cancers of bladder, lung and other organs, as well as with coronary heart disease and other conditions. |
| Cancer risk increase is associated with occupational exposure to asbestos, vinyl chloride, and other chemicals. |
| Radiation exposure is associated with an increase in risk of leukaemia and cancer. |
| Vaginal adenocarcinoma risk is increased in the daughters of women who received diethylstilboestrol during the pregnancy. |
| Endometrial cancer risk is increased in women taking oestrogens for postmenopausal symptoms. |
| Understanding of population trends in cancer survival. |

[a]Adapted from Gordis and Gold (1980).

Correspondence: M.P. Coleman, Medical Director, Thames Cancer Registry, 15 Cotswold Road, Sutton, Surrey SM2 5PY, UK.
Received 16 April 1992.

*Chair, George T. Duncan, c/o Committee on National Statistics, National Research Council, 2101 Constitution Avenue NW, Washington DC 20418, USA.

formation provided by persons or businesses for statistical purposes and the need to make publicly-collected data widely available for legitimate research and statistical uses.'

The UK Medical Research Council (MRC) recently revised its 1972 statement on the principles and standards of practice expected of researchers who use personal medical data (MRC, 1985). The MRC statement points out that doctors have a duty to build on the knowledge gained from treating patients; that medical advance often depends on pooling the experience of many doctors; and that 'advances made in this way are so important that the council believes that, provided every practical step has been taken to safeguard confidentiality and to ensure that no disadvantage, harm, distress or embarrassment is suffered by any individual as a consequence, there should be no impediment to the use of medical information in research'.

Effective cancer registration requires the collection and linkage of data about individuals with cancer, often from several different sources, over a number of years, from the diagnosis of the cancer until the death of the individual (Jensen *et al.*, 1991). It is essential that personal identifiers, including the individual's name and birthdate, be used in collecting these data, for the following reasons:

(a) to eliminate multiple counting of a single tumour (which would otherwise seriously inflate estimates of cancer occurrence in the population) by combining data about one individual from various sources at the time of initial cancer registration;

(b) to enable a tumour record to be completed with data obtained subsequently about recurrence, metastasis, new primary cancers, and death;

(c) to ensure that data about individuals in the registry are accurate, and can be readily corrected or updated;

(d) to enable production of cancer survival statistics. By following up patients registered with cancer, or by matching cancer registrations with death certificates, it is possible to assess the survival of *all* persons with cancer in a defined population. Survival from cancer in the population as a whole is frequently different from survival in selected series of patients, such as those recruited to clinical trials. Cancer survival can also be assessed by clinical stage or extent of spread at diagnosis, by type of treatment or by other variables recorded at cancer registration, such as socioeconomic category (Karjalainen & Pukkala, 1990). This in turn enables definition of prognostic variables and the design of early detection and treatment campaigns;

(e) to enable epidemiological studies of cancer to be carried out. The argument here closely parallels that underlying the notification of infectious diseases: it may only be possible to investigate infectious disease outbreaks effectively if identifiable individual case reports of the disease are available. To investigate possible causes of cancer, for example in case control studies, it is often necessary to contact patients to obtain information on past exposure to chemicals or other agents which may have caused their cancer.

The IACR statement notes that adequate safeguards for the individual's right to privacy can be obtained by adherence to an appropriate code of conduct in the operation of the cancer registry, just as in the operation of surveillance systems for notifiable sexually transmitted diseases and other infectious diseases, or in the operation of hospital records systems. Administrative restrictions on cancer notification – such as the requirement that patients give written or verbal consent for data about their cancer to be entered into a registry – produce uncontrollable selection bias and distortion of incidence data which seriously detract from the usefulness of the data collected. The position of the MRC is similar: 'consideration has been given to the question of whether [transfer of information from medical records for the purpose of research] requires the explicit consent of the patient. [ . . . ] The Council considers that, subject to scrupulous safeguards about confidentiality, information about patients can properly be available for medical research without their explicit consent, as it has been in the past' (MRC, 1985).

### Legal basis of cancer registration

The legal basis for cancer reporting during the period 1983–1987 is known for some 150 population-based cancer registries, in 50 countries, which have been invited to contribute to the next volume of Cancer Incidence in Five Continents, the international compendium of cancer incidence data (Parkin *et al.*, in preparation). The pattern is similar in all regions of the world (Table II). Just over half (55%) of the registries obtain reports on a purely voluntary basis from physicians, hospitals and other institutions treating cancer patients. For some 38% of registries, reporting is required either by laws of the state or by local administrative regulations, while for the remaining seven per cent of registries, the legal basis of reporting is mixed, some sources being required to report all diagnosed cancers, while others cooperate on a voluntary basis. Of the 105 registries included in volume V of Cancer Incidence in Five Continents, covering the period 1978–1982, 62 (59%) relied on voluntary reporting. Among registries which had begun operation since 1970, the proportion was slightly higher (64%) (Muir *et al.*, 1987). There is thus no evidence of any major recent shift toward mandatory reporting of cancer. In the EEC, 85% of registries still received cancer notifications on a voluntary basis in 1987 (Coleman & Démaret, 1988).

Where cancer is a disease that must be notified by law, the doctor or institution reporting the cancer to the registry will expect indemnity from legal action for breach of medical confidentiality; this may make it easier to encourage cooperation with cancer registration activities. Even where there is a legal requirement to report cancer, however, it is not easily enforceable, and there is little evidence that the mere existence of such a requirement can ensure completeness of registration, unless the necessary infrastructure for cancer registration is already in place. In the cancer registry of Finland, for example, reporting was voluntary until 1960, when it became compulsory: no difference was observed in the level of registration (Muir & Démaret, 1982). By contrast, the legal requirement to report cancers to the Turkish Ministry of Health since 1983 has resulted in less than a quarter of the expected number of cancer registrations, whereas in Denmark, cancer registration was voluntary from 1943 onwards, and had been largely complete for many years before notification of cancer became obligatory in 1987.

Laws designed to protect individual privacy may have the additional but unintended effect of making effective cancer registration impossible: for example, the number of cancers reported to the cancer registry in Hamburg, FRG, fell from 10,000 a year to just two cases in 1980–1981, owing to apprehension among physicians about possible legal consequences of reporting cancers in their patients to the registry, following a change in the rules governing transfer of such information between the registry and the Ministry of Health (Muir & Démaret, 1991). Since 1985, a special law has allowed physicians to report cases of cancer to the registry, but subject to the patient's consent (Thiele, 1990): it will be some time before the effects of this legislation can be evaluated.

If legislation is required, it should enable efficient and confidential reporting of cancer. Such a law should explicitly state that a cancer patient either should be (mandatory) or may legally be (voluntary) reported and registered in an identifiable manner, but that confidentiality must be maintained. The precise legal framework for cancer registration will obviously vary between states, but it should provide both a statement of the principles underlying confidentiality in the cancer registry, and a practical mechanism for ensuring that these principles are observed.

A few examples may serve to demonstrate the range of approaches adopted in relation to confidentiality and the outcome for cancer registration practice:

### *Finland*

The Finnish Cancer Registry operates under an agreement between the National Board of Health and the Cancer

**Table II** Legal basis of reporting to cancer registries,[a] by geographic region: number (per cent)[b] of registries

| | Voluntary | Compulsory By law | Compulsory By order | Mixed | Unknown | Total |
|---|---|---|---|---|---|---|
| Africa | 4 (100%) | – | – | – | – | 4 |
| S. America | 7 (58%) | 3 | – | 2 | 1 | 13 |
| N. America | 10 (45%) | 7 | 3 | 2 | 3 | 25 |
| Asia | 14 (54%) | 5 | 5 | 2 | – | 26 |
| Europe | 45 (57%) | 16 | 15 | 3 | 3 | 82 |
| Oceania | 2 (29%) | 4 | – | 1 | 4 | 11 |
| Total | 82 (55%) | 35 (23%) | 23 (15%) | 10 (7%) | 11 (–) | 161 |

[a]Cancer registries are those invited to contribute to *Cancer Incidence in Five Continents, vol. VI,* (in preparation). Data by courtesy of Ms Jean Powell and the editors of Cancer Incidence in Five Continents. [b]Percentage of registries for which the legal basis of registration is known.

Society of Finland, receiving data from hospitals, physicians and pathology laboratories. The registry is national, and began operating in 1953. Cancer notification is required by law and patients are not asked for consent to register their cancer. The registry data are used to produce routine statistics on cancer occurrence in the population, for reports to health authorities, to produce educational material both for the general public and for the medical profession, and for scientific research. Scientists needing access to information including personal identifiers for a given research project must first obtain permission from the Ministry of Health. Under the Finnish Law on Person Registers, registered patients do not have access to their data in the cancer registry because the files are used, under legal control, only for statistical and scientific purposes, and are not available for use by government or other agencies to make decisions concerning the individual (Hakulinen, 1990). The cancer registry is widely used for cancer research and its data are known to be of high quality. The registry has produced projections of cancer incidence, prevalence and mortality up to the year 2008 (Hakulinen *et al.*, 1989), and contributed to an analysis of cancer trends in the Nordic countries (Hakulinen *et al.*, 1986).

### *Federal Republic of Germany*

There are several regional registries and a national registry of childhood tumours. Patients must give consent for their data to be submitted to some of these registries. In Hamburg, although 98% of patients who are asked to consent to their cancer being recorded in the cancer registry do so, a large and unknown proportion of patients are not asked for their permission, and are thus not reported to the registry: 15–20% of cancer patients are not fully informed of their diagnosis, for reasons such as untreated cancer diagnosed in very old age, death in hospital after admission *in extremis*, and confusion (Thiele, 1990). In Saarland, the cancer registry ceased operating for a year in 1978 while the legal position of physicians reporting to the registry in relation to confidentiality was clarified, and an appropriate local law was passed (Muir & Dèmaret, 1982). The system under consideration for the cancer registry in Baden-Württemberg is based on reports received from doctors who first encrypt the patient's name with a computer program. The registry would use the encrypted code, instead of the name, to identify cases and to eliminate duplicates. The problems of identification (same patient coded differently and different patients coded identically) were examined in a pilot study: incidence was overestimated by up to 10%. Only 58.8% of 313 doctors surveyed found it acceptable that the patient's written consent be obtained before registration (Meisner *et al.*, 1990).

In 1983, the German Ministry for Youth, Family Affairs and Health commissioned a representative survey of 1,500 people to assess the opinions of the general public on cancer registration – possibly the only such survey ever carried out (Anon, 1983). Of those interviewed, 88% considered that the establishment of cancer registries was a valuable measure to fight cancer, and 78% agreed that their personal data should be reported and analysed in a cancer registry if they should develop cancer. Two thirds (66%) considered that the physician should obtain the patient's consent to report a cancer to the registry, but that the physician should not have to insist on this consent before reporting if doing so could result in additional psychological damage to the patient. It is of some interest that among the small proportion (12%) of persons giving an unfavourable opinion on cancer registration, one-half cited doubts about guarantees of data protection as their main reason.

The national cancer registry of the ex-German Democratic Republic, the largest in Europe, has recorded detailed information on cancers arising in a population of 17 million since 1953, and has been a particularly rich source of data for research (see for example Haas *et al.*, 1987). Since the reunification of Germany in 1990, however, registration has ceased and the data are not currently available for research (Sietmann, 1991), while a new legal basis is still being sought to permit both research with the existing data and continuation of cancer registration (Dickman, 1990).

### *France*

There are a number of regional population-based cancer registries, the earliest dating from 1975. Many produce valuable incidence data (Benhamou *et al.*, 1990) and are actively involved in research. All operate on the basis of voluntary reporting. Since 1988, seven cancer registers have been partially funded by the state, and eight more will be so funded from 1992. The law (article 378 of the Penal Code), however, does not currently allow a doctor to transmit identifiable medical data to a doctor not involved in the patient's treatment, and some doctors refuse to cooperate with cancer registries for this reason. A 1978 law on privacy and computerised databases also requires that persons must be informed of the use to be made of the data. A computerised register of AIDS patients has been created, however, in which the patient's name and birthdate are irreversibly encrypted at data entry; patients must give written consent for their data to be entered and can withdraw it at any time (Dorozyneski, 1988; Thirion *et al.*, 1988). Such a register can be used to measure incidence and survival, but it is subject to the same problems of duplicates and selection bias as in the German cancer registries, and cannot be used as the starting point for epidemiological studies requiring access to data on individuals. The paradox has been further highlighted by an epidemiological study, not of cancer, but of the genetics of manic depression, in which 30,000 people have been identified as being at risk of a treatable form of hereditary chronic glaucoma, genetically linked to manic depression, and which if untreated can lead to blindness. The individuals are known by name to the researchers, who are prevented by law from warning them (Nau, 1991). Legislation apparently in preparation would resolve this difficulty by providing adequate controls on individual privacy at the same time as facilitating epidemiological research.

**Guidelines on confidentiality**

Guidelines on confidentiality are required for efficient and acceptable registration of cancer. The main objectives of such guidelines are (Muir, 1991):

(a) to ensure the protection of confidentiality of data about individuals whose cancer is reported to the registry, so that information on registered persons cannot reach unauthorised third parties;

(b) to ensure that cancer registry data are of the best possible quality;

(c) to ensure that the best possible use is made of cancer registry data for the benefit of cancer patients, for cancer control in the population, and for medical research.

The existence of a set of guidelines will not by itself ensure either high quality or effective use of the data, but such guidelines define both the conditions under which high quality data may be collected, linked and stored in an ethical manner, and a framework to ensure safe and effective use of the data in a manner consistent with ethical guidelines (Last, 1990). A code of confidentiality will thus help to ensure that a proper balance can be struck between the individual's right of privacy and the right of the individual, and that of his or her fellow citizens, to benefit from the knowledge on cancer causation, prevention, treatment and survival that can be derived from cancer registration.

The existence of confidentiality guidelines, and evidence that they are respected in practice, may be necessary to reassure members of the public, especially cancer patients, that data stored in the cancer registry are treated according to standards of confidentiality that are at least as stringent as those used in the hospital or the physician's office, and that the use of these data for research purposes is covered by adequate safeguards. In some countries, such guidelines may need to be supported by law or regulation, and to specify sanctions in the event of breach of confidentiality, as well as a mechanism for monitoring the adequacy of data security procedures. Guidelines for confidentiality also provide registry directors with operational guidance, and protect the reporting physician or institution.

Some cancer registries expressed the need for model guidelines on confidentiality at the Hamburg meeting of IACR in 1990, even though most registries already have formal sets of rules for the maintenance of confidentiality of their data, often established in cooperation with the competent local authorities. The guidelines on confidentiality presented here (annex I) were originally prepared with the help of several IACR member cancer registries, a national vital statistics office and the EEC, and revised in the light of comments received from other IACR member registries. They have now been further revised and brought up to date, after circulation for comments to 325 cancer registries and individual members of IACR.

It should be stressed that these guidelines on confidentiality are not intended *en bloc* for adoption as a fixed set of rules by any particular cancer registry. The operating conditions of cancer registries vary greatly around the world, and a set of measures considered to be satisfactory in one cultural context and at a given period of time would prove inappropriate in another context: there is no simple, global solution to the problem posed by the maintenance of confidentiality. Instead, the guidelines are intended to outline some basic principles, and to provide a set of specific measures designed to ensure the preservation of confidentiality, from which a registry may select and reformulate, as necessary, those measures considered to be most useful in the preparation or revision of a local code of practice on confidentiality. The guidelines would need to be adapted to national or local circumstances; they should be used to complement rather than replace existing registry rules, and the resulting local code of practice on confidentiality would need to take account of local ethical practice.

An earlier version of the guidelines presented here has already been translated, adapted and incorporated in this way by a number of cancer registries and by a national AIDS register; it has also been used in the preparation of a training manual for cancer registry personnel in developing countries (IARC, in preparation). The earlier version was also adopted by the EEC Committee of Cancer Experts in May 1989 as the basis for its recommendation to the EEC Commission on guidelines for confidentiality in cancer registration.

The chief measures in the code of confidentiality for the operation of cancer registries are intended:

(a) to define what information is confidential;

(b) to specify measures for the security of data within the cancer registry;

(c) to require surveillance and periodic review of data security procedures;

(d) to define the conditions under which confidential data held by the registry can be released to approved medical research workers;

(e) to protect the reporting physician and institution.

In their testimony to the US Congress, Gordis and Gold (1980) laid special emphasis on the point that 'investigations of the natural history of disease and of the effectiveness of preventive and therapeutic interventions are of great potential benefit to society, but the conduct of such studies requires that, with proper safeguards, individually identifiable data from medical records continue to be made accessible for medical and epidemiological research'. In a number of countries, there are now pressing demands both for an absolute ban on any transfer of personal medical information *and* for rapid identification and control of health hazards to the public. The inherent conflict between these two positions (sometimes, with scant regard for logic, adopted simultaneously) can certainly be resolved, but only if governments can ensure that regulations designed to safeguard the privacy of the individual are framed so as to permit – indeed to encourage – well-conceived research. With this background, the guidelines for confidentiality in cancer registration presented here are intended to be of value for the data subject, the data supplier and the data user.

**References**

ANON (1983). *Most inhabitants are in favour of cancer registries.* Press release, 20 July 1983. Bundesministerium für Jugend, Familie und Gesundheit, Bonn, FRG.

BENHAMOU, E., LAPLANCHE, A., WARTELLE, M. & 7 others (1990). *Incidence des cancers en France, 1978–1982: estimation France entière.* INSERM: Paris.

COLEMAN, M. & DÉMARET, E. (1988). Cancer registration in the European Community. *Int. J. Cancer*, **42,** 339–345.

COLEMAN, M., WAHRENDORF, J. & DEMARET, E. (eds) (1992). *Directory of on-going research in cancer epidemiology*, IARC Scientific Publication 117. IARC: Lyon.

COMMISSION OF THE EUROPEAN COMMUNITIES (1984). *The confidentiality of medical records: the principles and practice of protection in a research-dependent environment.* EUR 9471 EN. CEC: Luxembourg.

COUNCIL OF EUROPE (1981). *Convention for the protection of individuals with regard to automatic processing of personal data.* European Treaty Series no 108. Council of Europe: Strasbourg.

DICKMAN, S. (1990). Valuable cancer registry. *Nature*, **346,** 306.

DOROZYNESKI, A. (1988). French AIDS register. *Brit. Med. J.*, **297,** 1003–1004.

GORDIS, L. & GOLD, E. (1980). Privacy, confidentiality and the use of medical records in research. *Science*, **207,** 153–156.

HAAS, J.F., KITTELMANN, B., MEHNERT, W.H. STANECZEK, W., MOHNER, M., KALDOR, J.M. & DAY, N.E. (1987). Risk of leukaemia in ovarian tumour and breast cancer patients following treatment by cyclophosphamide. *Brit. J. Cancer*, **55,** 213–218.

HAKULINEN, T., ANDERSEN, AA., MALKER, B., PUKKALA, E., SCHOU, G. & TULINIUS, H. (1986). Trends in cancer incidence in the Nordic countries. *Acta. Path. Microbiol. Immunol. Scand. Sect. A*, **94**, suppl 288.

HAKULINEN, T., KENWARD, M., LUOSTARINEN, T. & 4 others (1989). Cancer in Finland in 1954–2008: incidence, mortality and prevalence by region. Finnish Cancer Registry: Helsinki.

HAKULINEN, T. (1990). *Laws, by-laws and administrative actions: coping with confidentiality at the Finnish Cancer Registry*. Conference of the International Association of Cancer Registries, Hamburg, FRG, August 1990, session C2. (Conference abstract).

HEASMAN, M.A. (1982). Data protection and community medicine. *Comm. Med.*, **4**, 169–172.

JENSEN, O.M. & STORM, H.H. (1991). Purposes and uses of cancer registration. In Jensen, O.M., Parkin, D.M., MacLennan, R., Muir, C.S. & Skeet, R.G. (eds). *Cancer registration: principles and methods*. IARC Scientific Publication 95, IARC: Lyon, pp. 7–21.

JENSEN, O.M., PARKIN, D.M., MACLENNAN, R., MUIR, C.S. & SKEET, R.G. (1991). *Cancer registration: principles and methods*. IARC Scientific Publication 95. IARC: Lyon.

KARJALAINEN, S. & PUKKALA, E. (1990). Social class as a prognostic factor in breast cancer survival. *Cancer*, **66**, 819–826.

LAST, J.M. (1990). Guidelines on ethics for epidemiologists. *Int. J. Epidemiol.*, **19**, 226-229.

MEISNER, C., PIETSCH-BREITFELD, B. & SELBMANN, H.K. (1990). *The planned cancer registry in Baden-Württemberg (FRG): between medical secrecy, protection of data privacy and epidemiological research*. Conference of the International Association of Cancer Registries, Hamburg, FRG, August 1990, session C2. (Conference abstract).

MEDICAL RESEARCH COUNCIL. (1985). Responsibility in the use of personal medical information for research: principles and guide to practice. *Brit. Med. J.*, **290**, 1120–1124.

MUIR, C.S. & DÉMARET, E. (1982). *Some current problems in cancer registration*. Internal Technical Report 82/003. IARC: Lyon.

MUIR, C.S. & DÉMARET, E. (1991). Cancer registration: legal aspects and confidentiality. In Jensen, O.M., Parkin, D.M., MacLennan, R., Muir, C.S. & Skeet, R.G. (eds). *Cancer registration: principles and methods*. IARC Scientific Publication 95. IARC, Lyon. pp. 199–207. IARC: Lyon.

MUIR, C.S., WATERHOUSE, J., MACK, T., POWELL, J. & WHELAN, S. (eds). (1987). *Cancer incidence in five continents*, Vol. V. IARC Scientific Publication 88. IARC: Lyon.

NAU, J.-Y. (1991). *Les cécités de la loi (The law that's blind to progress)*. Le Monde, 3 April, pp. 17–18.

PARKIN, D.M., WAGNER, G. & MUIR, C.S (eds). (1985). *The role of the registry in cancer control*. IARC Scientific Publication 66. IARC: Lyon.

THIELE, W. (1990). *Data protection in the Hamburg Cancer Registry*. Conference of the International Association of Cancer Registries, Hamburg, FRG, August 1990, session C2. (Conference abstract).

THIRION, X., SAMBUC, R. & SAN MARCO, J.L. (1988). L'anonymat dans les enquêtes épidémiologiques: étude et mise en oeuvre d'une nouvelle méthode. *Rev. Epidém. et Santé Publ.*, **36**, 36–42.

SIETMANN, R. (1991). East German cancer data: a benefit of big brother? *Science*, **252**, 915.

### Appendix

### Guidelines on confidentiality in the Cancer Registry

### International Association of Cancer Registries

1. Purpose of guidelines on confidentiality in the cancer registry
   - 1.1 Aims of document
   - 1.2 Main components
   - 1.3 Background
   - 1.4 Right to privacy
   - 1.5 Use of guidelines
2. Definitions
   - 2.1 Cancer
   - 2.2 Cancer registry
   - 2.3 Cancer registration
   - 2.4 Confidential data
   - 2.5 Treating physician
   - 2.6 Security
3. Role of the cancer registry
   - 3.1 Function of cancer registry
   - 3.2 Legal basis of cancer registration
   - 3.3 Sources of information
   - 3.4 Use of cancer registry data
     - 3.4.1 Clinical use of identifiable data
     - 3.4.2 Transfer of identifiable data for registration puposes
     - 3.4.3 Use of identifiable data for research
       - (a) Studies of the causes of cancer
       - (b) Evaluation of screening
       - (c) Evaluation of survival from cancer
     - 3.4.4 Use of aggregate data
       - (a) Research
       - (b) Health care planning
4. Principles of confidentiality
   - 4.1 Underlying concept of medical confidentiality
   - 4.2 Sharing of confidential clinical information
   - 4.3 Legal protection of data suppliers
   - 4.4 Confidentiality and utility
   - 4.5 Scope of confidentiality measures
   - 4.6 Confidentiality of data on deceased persons
   - 4.7 Indirectly identifiable data
   - 4.8 Methods of data storage and transmission
5. Measures for data security
   - 5.1 Responsibility
   - 5.2 Oath of secrecy
   - 5.3 Physical access to the registry
   - 5.4 Persons with access to registry
   - 5.5 Persons with access to confidential data
   - 5.6 Display of reminders
   - 5.7 Active registration
   - 5.8 Incomplete data
     - 5.8.1 Cases identified from death certificates
     - 5.8.2 Matching of data files
   - 5.9 Transmission of information
     - 5.9.1 Postal services
     - 5.9.2 Magnetic or electronic media
     - 5.9.3 Electronic transmission
   - 5.10 Use of telephone
   - 5.11 Use of computer
     - 5.11.1 Data entry
     - 5.11.2 Use of database
     - 5.11.3 Demonstrations
     - 5.11.4 Backup
   - 5.12 Unauthorised access to computer system
   - 5.13 Paper storage
   - 5.14 Disposal of paper records
   - 5.15 Review of security procedures
6. Release of data
   - 6.1 Responsibility for data release
   - 6.2 Limitations on data release
   - 6.3 Release of identifiable data for clinical purposes
   - 6.4 Release of identifiable data for research
   - 6.5 Release of aggregate data
   - 6.6 Provision of data to individuals
   - 6.7 Studies involving several registries
   - 6.8 News media

6.9 Conditions for release of data
6.10 Guidelines for release of data
6.11 Cessation of registry activity

7. Summary of conclusions and recommendations

7.1 Principles of confidentiality and the role of the cancer registry
7.2 Measures for data security
7.3 Release of registry data

### 1. Purpose of guidelines on confidentiality in the cancer registry

1.1 *Aims of document*

The aims of this document, and of the accompanying article, are:

(a) to advance the need for a code of conduct in the maintenance of confidentiality in cancer registries;
(b) to define the aims of maintaining confidentiality in cancer registries;
(c) to set out the principles of confidentiality;
(d) to advance guidelines for the preservation of confidentiality; and
(e) to advance guidelines for the use and release of registry data in accordance with these principles.

1.2 *Main components*

These guidelines are intended:
(a) to define what information is confidential;
(b) to specify measures for the security of data within the cancer registry;
(c) to propose surveillance and periodic review of data security procedures;
(d) to define the conditions under which data held by the registry can be released and the persons to whom it can be released (data users); and
(e) to protect the reporting physician and institution (data suppliers).

1.3 *Background*

The background to these guidelines is presented in the accompanying article, which should be read in conjunction with these guidelines.

1.4 *Right to privacy*
Guidelines for the maintenance of confidentiality are needed primarily to provide adequate safeguards for the individual's right to privacy, so that identifiable information on persons registered with cancer does not reach unauthorised third parties, while at the same time preserving the right of the individual, and that of his or her fellow citizens, to benefit from the knowledge on cancer causation, prevention, treatment and survival that can be obtained from cancer registration and research.

1.5 *Use of guidelines*

Guidelines for the maintenance of confidentiality are also needed, however, to help ensure that cancer registry data are of the best possible quality, and that the best possible use is made of the data, both for the benefit of cancer patients, for cancer control in the population, and for medical research. In order for cancer registry data to be of value for clinical, statistical and research purposes, the data recorded must be as complete, accurate and reliable as prevailing circumstances permit. These standards of quality can only be achieved if both the public and the physicians and institutions treating cancer patients are confident that the data required are necessary for the objectives of cancer registration and medical research, and that confidential data will be adequately safeguarded.

These guidelines are not intended to be adopted *en bloc* or without modification as a fixed set of procedures for the maintenance of confidentiality in any particular cancer registry. Rather they are intended to present the basic principles of confidentiality, and to provide a set of measures from which a registry may select and reformulate, as appropriate, those measures considered to be most useful in the preparation or revision of a local code of practice on confidentiality.

The applicability of these guidelines will be kept under review by the IACR, and amendments made as necessary.

### 2. Definitions

2.1 *Cancer*
The term 'cancer' is used in this document to imply all malignant neoplasms, as defined in the International Classification of Diseases for Oncology, second edition (Percy *et al.*, 1990).

2.2 *Cancer registry*
A cancer registry may be defined as an organisation for the collection, storage, analysis and interpretation of data on persons with cancer. Cancer registries which limit their aims to recording particulars of cancer cases seen in a given hospital or group of hospitals are said to be hospital-based: such registries are frequently located in the hospital records department. Cancer registries which aim to register details of every cancer that occurs in a defined population, usually those persons habitually resident within the boundaries of a defined territory or geographic region, are said to be population-based. Such registries are often based within a hospital, but may also be located in a separate building or institution. Population-based registries may be general (recording all tumours) or specialised (restricted to a given site-group or age-group).

2.3 *Cancer registration*
Cancer registration is the process of continuing, systematic collection of data on the characteristics of all cancers and of the persons diagnosed with cancer, and is the basic activity of a cancer registry.

2.4 *Confidential data*
For the purposes of this document, any data collected and stored by a cancer registry which could permit the identification of an individual patient (data subject) or, in relation to a particular data subject, of an individual physician or institution (data supplier) are considered to be confidential. Data which could permit identification (identifiable data) include names, address, full date of birth, date of death, and unique reference numbers (e.g. national identity numbers).

2.5 *Treating physician*
For the purposes of this document, the treating physician may be defined as the doctor primarily responsible for the patient's cancer treatment; or a doctor to whom the patients has been referred for additional investigation or treatment; or the patient's usual physician; the medical director of the institution where the treating physician is or was employed when treating the patient in question may also act on behalf of the physician.

2.6 *Security* (or data protection) denotes the measures taken to ensure the maintenance of confidentiality of the registry data, whether stored on paper, microfilm, microfiche or magnetic media.

### 3. Role of the cancer registry

3.1 *Function of cancer registry*
The cancer registry plays a central role in the systematic collection, recording and analysis of data relating to individuals with cancer. For each such person 'it is the function of the registry to record, as fully and as accurately as may be possible, both a clinical description of the extent of the disease and also information which will identify the patient, the tumour, the hospital and the clinicians involved with the case. When these data are combined with additional information describing treatment and subsequent progress (routine follow-up), in which recurrences, metastases and further treatment are included, terminating with the date and cause of death (whether from cancer or not), a very full and invaluable data bank can be created' (Waterhouse, 1978).

3.2 *Legal basis of registration*
Cancer registration may be based on compulsory or voluntary notification of cancer patients to the registry. Compulsory registration may arise from legislation passed by a parliament or elected legislative body (primary legislation), or from an administrative order issued under the aegis of a statutory agency such as the Ministry of Health or a provincial health authority. Some cancer registries may obtain both voluntary and compulsory notifications, depending on the source of information: in some areas, for example, pathologists report voluntarily, while the Vital Statistics Office is legally required to do so; in others, pathologists are legally required to report cancers to the registry, while treating physicians report voluntarily.

3.3 *Sources of information*
Notifications of cancer may be derived from many sources, such as the treating physician, surgeon, radiologist or radiotherapist; hospital admissions and records departments, the hospital discharge report, or laboratories of pathology, cytology, haematology or biochemistry; medical records of social security systems; and coroners and vital statistics offices (death certificates). Notifications may be submitted on paper records or, increasingly, on magnetic media. In some areas, registry employees may visit the source of information to obtain notifications (active registration), while in others the sources of information may submit these directly to the registry (passive registration). Many registries use both active and passive methods of registration.

3.4 *Use of cancer registry data*
The purposes for which data collected by the cancer registry are used should be clearly defined. Cancer registries are important sources of data, both for clinical purposes and for research intended to advance understanding of the causes, occurrence and outcome of cancer. Such data may be either identifiable or aggregate (anonymous), depending on the nature of the research. Some examples of the use of cancer registry data in relation to confidentiality are outlined here: the list is not intended to be exhaustive, but to identify major categories of use.

3.4.1 *Clinical use of identifiable data*

Clinical use of identifiable data relating to patients registered with cancer arises in the context of their diagnosis, treatment and follow-up by the treating physician(s). Availability to the treating physician of identifiable data may enable duplication of diagnostic procedures to be avoided, may facilitate exchange of information between treating physicians, and may assist the physician to evaluate the outcome of treatment in individual patients or in groups of patients. Identifiable data required for such clinical purposes may therefore be provided to the treating physician on request, and in accordance with the procedures outlined in section 6, in order to assist the physician in the management of his or her patients with cancer.

3.4.2 *Transfer of identifiable data for registration purposes*

In two circumstances, registries may need to transfer identifiable data to other cancer registries for the purposes of complete registration. The first case involves a tumour diagnosed in a person who proves to be resident in the territory of another, usually adjacent, registry. The second case involves regional registries which contribute data to a larger or national registry, or specialised registries which also contribute data to a general population-based registry. In each case, data may be transferred for the purposes of complete and accurate registration, provided that the recipient registry adheres to comparable standards of confidentiality.

3.4.3 *Use of identifiable data for research*

(a) Studies of the causes of cancer

Case-control and cohort studies help in identifying the causes of cancer. Both types of study require information about individuals with cancer. In a cohort study, for example, linking the cohort members against the cancer registry files (or against a file of death certificates) enables cancers arising in the cohort to be detected. This has proved a highly efficient, economical and confidential method of detecting risk. Such linkages may be manual, computerised or both, and while linkage always requires knowledge of the identity of individuals with cancer, the resulting publications always present anonymous or aggregated data. Registries are frequently used as a source of cases (and sometimes also of controls) for case-control studies: the value of these studies for identifying risk factors is enhanced by the availability of a representative sample of tumours diagnosed in the population.

(b) Evaluation of screening

Cancer registries play a major role in the evaluation of screening programmes, by providing information to enable the assessment of whether, in comparison to an unscreened population, invasive cancer, e.g. of uterine cervix or breast, develops less frequently and mortality decreases in a screened population or subgroup. This requires comparison of lists of individuals with cancer detected by the screening programme with cancer registry files. The cancer registry may thus be essential for adequate evaluation of a population-based cancer screening programme, providing information not available in any other way.

(c) Evaluation of survival from cancer

By matching death certificates to cancer notifications received by the registry, it is possible to assess the survival of *all* persons with cancer in a defined population. Survival from cancer in the population as a whole is frequently quite different from that reported for selected series of patients (e.g. in clinical trials). Such data may be used to evaluate the extent and speed with which new or improved cancer treatments are incorporated into routine clinical practice. It is also possible to assess population survival for a given cancer by the extent of spread at diagnosis, or by type of treatment. This type of research is only possible if the registry can link identifiable cancer registrations with death certificates; such evaluation of cancer survival is now routine practice in many registries.

3.4.4 *Use of aggregate data*

(a) Research

One of the most important contributions of the cancer registry is to provide current data on the incidence of various types of cancer, and on variations in incidence by age, sex, place of birth, occupation, ethnic group, etc. These data can also be used to study differences in histological types and between urban and rural areas, and to examine trends in incidence over time. Only aggregate, anonymous data are used in such studies.

(b) Health care planning

Information provided by the cancer registry on the numbers of cancer patients can help health authorities in various ways, including long-term planning for the provision of medical facilities and the training of health care professionals; establishment of priorities and programmes for cancer control; evaluation of the effects of intervention; and estimation of the numbers of cancer patients in the future (projections). For all these purposes the identity of individual cancer patients is neither needed nor provided: only aggregate data are used.

### 4. Principles of confidentiality

4.1 *Underlying concept of medical confidentiality*

The set of principles outlined below relates to the preservation of confidentiality in connection with or during the process of collection, storage, use, and transmission of identifiable data by the cancer registry. A cancer registry must maintain the same standards of confidentiality in handling identifiable data as customarily apply to the doctor-patient relationship; this obligation extends indefinitely, even after the death of the patient.

These guidelines are intended to help ensure the confidentiality of data about individuals whose cancer is reported to the registry, so that information on registered persons cannot reach unauthorised third parties.

4.2 *Sharing of confidential clinical information*

For serious diseases such as cancer, 'in modern medical practice, the doctor can seldom be the sole confidant, since effective care involves others, both medical and non-medical, technical and clerical, who provide services and manage the health care institutions' (Medical Research Council, 1985). Despite this essential dispersion of confidential information within the clinical team, ultimate responsibility for the maintenance of confidentiality remains with the treating physician. The treating physician who provides information to a cancer registry about a patient with cancer therefore has the right to expect that the registry observes strict rules of confidentiality (see section 5.1).

4.3 *Legal protection of data suppliers*

Unless cancer is a disease which must be notified to a cancer registry by virtue of a law or administrative order, the data recorded by the cancer registry are supplied on a voluntary basis by the physician or institution. In some countries, therefore, it may be necessary for the registry to ensure that there is at least legal authority for physicians to report cancer, in order to protect data suppliers from legal action for breach of confidentiality in submitting identifiable data to the cancer registry.

4.4 *Confidentiality and utility*

Effective operation of the cancer registry depends on the continuous supply of confidential information from several sources, notably clinicians, pathologists and vital statistics offices. These data suppliers can only be expected to continue to provide such information if the cancer registry can be trusted to maintain confidentiality and to make good use of the data. Data suppliers will therefore need to be satisfied that the registry adheres to an adequate set of guidelines on confidentiality, and that data of high quality are being collected and used for the benefit of cancer patients and cancer research.

4.5 *Scope of confidentiality measures*

Maintenance of the confidentiality of identifiable data held by the cancer registry should extend beyond information on cancer patients and those notifying them (data subjects and data suppliers), to include identifiable data from medical records, census data, interview records, death certificates and lists of members of industrial cohorts or other study populations which may be stored in or provided to the cancer registry as part of its routine operations or for research projects.

4.6 *Confidentiality of data on deceased persons*

Data on deceased persons held in the cancer registry should be subject to the same procedures for confidentiality as data on living persons, even though death certificates or related information may be available from other sources, or in the public domain.

4.7 *Indirectly identifiable data*

Individual records from which names and address have been removed, but from which it might still be possible to identify an individual indirectly by use of the remaining data, e.g. an identity number, should also be subject to the measures for preservation of confidentiality in the cancer registry.

4.8 *Methods of data storage and transmission*

Guidelines for the maintenance of confidentiality are applicable not only to the storage of identifiable data on computers, but also to the storage of such data in the form of paper records, microfilm, microfiche and magnetic media, and their transport or transmission by registry personnel in any of these formats. The procedures involved may differ, but the underlying principle is the same.

### 5. Measures for data security

5.1 *Responsibility*

The Director of the cancer registry is responsible for maintaining the confidentiality of identifiable data. The Director must ensure that all the registry staff are aware of their individual responsibilities with respect to confidentiality, and that the security measures adopted by the registry are known and adhered to by all staff. Depending on the country, the Director's responsibility for data security may need to be defined in appropriate legislation, or by administrative order, or in other documents establishing the status and function of the registry.

5.2 *Oath of secrecy*

Duly trained and specialised staff should be appointed to run the cancer registry in accordance with its aims and rules of operation. It is recommended that, as part of their contract of employment or conditions of service, each member of the registry staff be required to sign a special declaration to the effect that they will not disclose confidential information held by the cancer registry to an unauthorised person at any time, or to any other person except as permitted within the context of the registry's guidelines on confidentiality. The terms of the contract of employment should make it clear that breach of this undertaking will result in disciplinary action which may involve a fine or dismissal. This declaration of secrecy shall remain in effect even after the staff member ceases to be employed in the cancer registry.

5.3 *Physical access to the registry*

Suitable locks and alarm systems should be installed to control physical access to the registry. Consideration should be given to the use of special locks with entry codes, or electronic methods of controlling access, and to the maintenance of a record of persons other than staff members who enter the registry.

5.4 *Persons with access to registry*

Unauthorised access to the cancer registry should be prevented. The Director of the registry should maintain an up-to-date list of all persons authorised to enter the registry.

5.5 *Persons with access to confidential data*

The Director of the registry should maintain an up-to-date list of registry staff members indicating the type of data to which each of them has access (with the corresponding level of computer security, if relevant).

5.6 *Display of reminders*

It is recommended that notices reminding staff of the need to maintain confidentiality be prominently displayed within the registry.

5.7 *Active registration*

Registry staff assigned to collect information at source (active registration) are responsible for maintaining the confidentiality not only of identifiable data they may collect on persons with cancer for the registry, but also of other information of a confidential nature which they may read or hear at the source.

Cancer registries using active methods of registration should give consideration (a) to providing staff with a lockable attaché-case for the transport of confidential information; (b) to advising staff on other measures to avoid accidental loss of such material; and (c) to providing staff with suitable means of identification as an employee of the cancer registry. The identity of such staff should be made known to the relevant person(s) at each of the sources which they visit to collect information for the registry, and where possible, changes in personnel should be notified to these sources in advance.

5.8 *Incomplete data*

If it is necessary for the registry to request additional information from a source concerning a particular registration, for example to check an address or date of diagnosis, this should be done by sending a confidential enquiry to a named individual at the source concerned. One technique used by certain registries is to arrange for the computer to generate standardised requests for missing data items which are then mailed under confidential cover, or electronically.

5.8.1 *Cases identified from death certificates*

Comparison of registry files with death certificates may reveal a death certified as due to a cancer not apparently registered in life. Registries which seek further information about cases first identified from a death certificate should request information from the certifying physician or the vital statistics office, according to local circumstances; if no further information can be obtained, the registry must decide whether to register such cases solely on the basis of the death certificate ('death certificate only', or DCO).

5.8.2 *Matching of data files*

The registry files may need to be matched against other computer files, either to provide missing data items or for the purposes of research. If it is necessary for such matching to be undertaken outside the registry, e.g. in a vital statistics office or on an external computer, the registry should first ensure that the confidentiality of its records will be preserved by the agency receiving the registry data.

5.9 *Transmission of information*

Authority to transmit identifiable data from the registry, whether by mail (in paper or machine-readable form), by telephone or by electronic means, should be obtained before transmission from the Director or other nominated staff member to whom specific responsibility for such transmission has been delegated.

5.9.1 *Postal services*

Cancer registries may need to receive and send cancer notifications and other confidential data by post in written or printed form. Consideration should be given (a) to the use of registered post or other forms of recorded acceptance and delivery by the postal service; (b) to the possibility of sending lists of names and other identifiable data separately from any other information being transmitted; (c) to the use of double envelopes, the external envelope giving a general address, and the internal envelope being marked for opening only by a named individual. If regular postal services are considered unreliable, use of a courier service may be envisaged: such a courier service should provide a written undertaking to maintain the security of cancer registry material.

5.9.2 *Magnetic or electronic media*

When identifiable data are sent by post on magnetic tapes, diskettes or in other machine-readable form, suitable precautions should be taken to ensure the physical security of the material in transit (as in the preceding paragraph). In addition, steps should be taken to ensure that the data cannot easily be read by an unauthorised person. Among the precautions which might be taken are:

(a) encrypting of names at various levels of complexity;
(b) sending the tape, diskette (etc) containing names, address and other identifiable data separately from the media containing tumour-related or other data, using a link number to enable reconstitution of the record by the intended recipient, and giving maximum security to the media containing identifiable data.

5.9.3 *Electronic transmission*

Many cancer registries use computers which are 'stand-alone', i.e. are not electronically linked to any other computer; these are usually owned by or dedicated solely to the cancer registry, and sited on its premises. Such computers cannot be used to transmit data electronically. Computer systems capable of electronic transmission may be classified into three groups:
(a) microcomputers with a 'modem', capable of transmitting and receiving via a standard telephone line;
(b) larger installations, still dedicated to the registry but with a fixed connection to an electronic communication network; and
(c) installations which the cancer registry shares with other users, such as a health ministry or university department computer.
As information systems evolve, an increasing amount of data is being sent to cancer registries by electronic means, usually via telephone lines dedicated to such purposes (i.e. not used for speech). A registry may also send its data for storage on a computer shared with another agency by this means (class (c) above).

Consideration should be given to measures such as encrypting names, and sending identifiable data separately from tumour and other data, with link numbers or codes, as outlined in the preceding section. Such measures would be applicable to data sent electronically either to a computer or to a telefax.

5.10 *Use of telephone*

It must be clearly recognised that use of the telephone, although convenient, may give rise to a breach of confidentiality.

No identifiable data or confidential information of any kind should be given to telephone callers by registry staff unless the caller is already an authorised recipient of such information, can give proof of identity and can justify the need to have the information by telephone rather than in writing. Identity should be requested by asking the caller to give his or her name, telephone number, address and position or title. After verification, a member of registry staff authorised to divulge such information should do so by calling back the person requesting it.

The need for the registry to pass identifiable information to external callers by telephone should be infrequent. One example might be where a clinician requests specific information concerning a patient in the context of managing that patient's disease.

5.11 *Use of computer*

Various physical and electronic measures are available to prevent unauthorised access to information held on the computer. The electronic measures are subject to rapid evolution, and will only be discussed in general terms here.

5.11.1 *Data entry*

Where feasible, the computer terminal(s) used for data entry may be placed in a separate room, access to which is restricted.

Other precautions may include:

(a) use of user names and passwords which do not appear on the screen when typed;
(b) change of passwords at intervals;
(c) automatic logging by the computer of all successful and unsuccessful attempts to enter the system, with regular checks of this log against written records of sessions spent at the terminal by the authorised users.

5.11.2 *Use of database*

Registry staff and other authorised users of its computer database should also be subject to the relevant precautions outlined in the preceding paragraph. Where possible, consideration should also be given to providing different levels of access to the database, such that only users authorised to gain access to identifiable data can do so.

5.11.3 *Demonstrations*

Cancer registries are frequently asked to demonstrate their computer system. When such demonstrations are given, it is recommended that the data used be fictitious or anonymised, and that the screen displays be labelled 'Demonstration', so that visitors are aware of this. For such purposes, it may also be possible to have a separate 'dummy' dataset, accessed by a special password and used only for demonstrations. The data used might be real, but with the names and addresses removed, scrambled or substituted with fictitious ones.

5.11.4 *Backup*

The computer database will usually be backed up to tapes or diskettes on a regular basis (daily, weekly, etc), for physical storage outside the registry as a precaution to avoid loss of the entire database in the event of extensive damage to the registry by fire or other hazards. It is advisable that such copies of the electronic database be stored in a locked fire-proof safe or an equivalent secure location, if possible on the premises of an agency such as a professional medical society, health ministry, medical ethical committee or legal authority.

5.12 *Unauthorised access to computer system*

It must be recognised that some persons may attempt to gain remote electronic access to computer systems, often to show that this is possible rather than to examine the data. It is unlikely that registries using computer systems to which remote electronic access is possible can provide absolute protection against any such attempt at reasonable cost. The level of security built in to such systems should at least be capable of foiling casual attempts to gain unauthorised access. Consideration should also be given to obtaining expert advice on enhancing the electronic security of such computer systems; this aspect of security should be regularly reviewed. Although it may not always be possible, in this context it is preferable that the cancer registry have an isolated data processing system.

5.13 *Paper storage*

Electronic methods of storage of identifiable data in cancer registries are now almost universal, but most registries also store a considerable amount of data on paper. Such material may include cancer registry notification forms, medical records, copies of pathology reports, copies of death certificates, etc. It is usually not practicable to keep names and other identifiable data separately from such material. Unlike data in computers, which can be readily protected against all but the most determined intruder, paper records are accessible to casual inspection, and require no special expertise to gain access. Consideration must therefore be given to keeping this material as secure as possible. Specific measures that may be considered include:
(a) defining who has access to the registry premises;
(b) defining which members of staff have access to the room where these materials are kept;
(c) providing lockable storage cabinets in which all confidential materials should be stored at the end of a working session; and
(d) ensuring that persons not authorised to do so (e.g. cleaning personnel) are not able to scrutinise paper or other physical records containing confidential data.

5.14 *Disposal of paper records*
A suitable policy should be developed for the safe disposal of waste paper and other physical records containing identifiable data. As in certain hospital records departments, many registries microfilm the paper records of registered persons at a certain interval after death, and destroy the paper record. Such destruction would normally involve shredding. Where the volume of confidential records to be destroyed is large, it may be necessary to employ specialised services for the safe disposal of confidential waste.

5.15 *Review of security procedures*
It is recommended that cancer registries undertake formal review of their security procedures at appropriate intervals. It may be helpful to recruit the services of specialist advisers to ensure that the registry's procedures for the maintenance of confidentiality are up-to-date, and cover all aspects of the registry's operations.

### 6. Release of data

One aim of all cancer registries is to make data usefully accessible for clinical purposes, for research and for use in health care planning (section 3.4). Some of these uses involve the release of identifiable data on individuals registered with cancer. Whilst adhering to its guidelines on the maintenance of confidentiality, therefore, the registry must also develop procedures to deal with requests for the release of confidential data.

6.1 *Responsibility for data release*

The Director of the registry would normally decide whether a request for information including confidential data on individuals can be met within the registry's guidelines, or whether wider consultation is required, for example through a scientific committee, internal review board or ethics committee competent to deal with such requests.

6.2 *Limitations on data release*

In the absence of written consent from all the parties concerned, a cancer registry should not release identifiable data either about a registered person (data subject) or, in relation to such a person, about a treating physician or institution (data supplier), for any purpose other than those outlined for clinical and research purposes (section 3.4). Enquiries may be received for identifiable data concerning individuals (who may or may not have a cancer recorded at the registry) from agencies such as pension schemes, health care cost reimbursement schemes or industrial disease compensation panels, or in the context of medical examination for life assurance or employment. Such requests for information, even from physicians, should be refused in terms which do not indicate whether the individual is or is not registered, and the enquirer should be asked to obtain information directly from the subject or the subject's treating physician.

6.3 *Release of identifiable data for clinical purposes*

Physicians who request data in the context of treating a patient registered with cancer should be given unrestricted access to the registry's data for that patient.

6.4 *Release of identifiable data for research*

The cancer registry should consider the release of confidential data only if the request is received in writing, if the nature of the request falls within the accepted range of uses of registry data, and if the request meets the registry's requirements for safeguarding the confidentiality of its data. The request should therefore be expected to include:
(a) the exact purpose for which the data are needed;
(b) the nature of the information required, and a justification of the need for confidential data; and
(c) the name and position of the person(s) who would have access to the confidential data; and
(d) the period of time for which the data would be used, and the way in which the data would be disposed of after the elapse of this period.

6.5 *Release of aggregate data*

Aggregate data in tabular or comparable formats (e.g. the numbers of persons registered with a given cancer by age, sex, year, etc) would not normally be subject to constraints on release in relation to confidentiality. For small geographic areas, however, tables containing cells with very few entries could in theory make it possible for individuals to be identified, and the registry should consider suitable measures to avoid this.

6.6 *Provision of data to individuals*

The cancer registry should not in general inform individuals whether or not there are data about them in the registry, unless required to do so by law: such requests should be referred to the treating physician or to the person responsible for data protection in the treating institution. It is preferable that any information about individuals be divulged through the treating physician, rather than directly to the person concerned.

6.7 *Studies involving several registries*

Research projects involving the provision of data about individuals from many cancer registries, sometimes in different countries, have provided valuable information about cancer risk. Whilst it may be necessary for individuals to be identifiable within the context of such studies, identifiable data should not normally be transmitted to other registries or countries. Each subject may be allocated a suitable number by which his or her record can be traced in the cancer registry of origin by registry staff, for data verification within the study. This number can then be used instead of the subject's identity in data files contributed to the study coordinating centre.

When the study design requires that identifiable data can be transmitted across registry or national borders, and if legislation permits, then such transferred data should remain subject to the same rules of confidentiality as in the registry of origin. Cancer registries participating in such studies should satisfy themselves that their data will be treated accordingly. It may be advisable that data for such studies be transferred to a suitable independent research agency or a reserach centre recognised by the World Health Organization.

6.8 *News media*

Cancer registries are frequently approached by the press for information on cancer. It is recommended that all such enquiries be referred to the Director or other nominated staff member to whom specific responsibility for dealing with the press has been delegated.

6.9 *Conditions for release of data*

The Director of the registry should obtain satisfactory evidence that the intended recipient of data requested for research purposes will:
(a) observe the same principles of confidentiality as are observed by the staff of the cancer registry;
(b) comply with all restrictions imposed by the registry on the use of the data, in particular that they will not be used for purposes other than those agreed at the time of provision of the data, and that they will not be communicated to other parties;
(c) not contact registered persons (or relatives of persons) whose identities have been provided in confidence by the cancer registry (e.g. for research based on interview) *except* if written authorisation to do this has first been obtained from the treating physician in each case: in some countries the project may also need to be approved by an ethical committee;
(d) ensure that any publication of the results of research will not enable any individual to be identified; and
(e) return or destroy in an approved manner all data which are no longer required for the purpose specified in the request.

6.10 *Guidelines for release of data*

It is recommended that registries consider preparing a document which sets out the procedures and criteria applicable to the release of their data, especially the release of identifiable data for research. Such a document could be provided to researchers requesting identifiable data.

Recipients of identifiable data approved under these procedures should also be asked to provide signed commitments to respect the confidentiality of such data and to adhere to the registry's guidelines for the use of the data, including destruction or return of the data on completion of the research.

6.11 *Cessation of cancer registration*

Each cancer registry should develop a policy for the actions to be taken in the event that the registry ceases operation. Consideration should be given to methods of storage of the registry database in an archive, so as to preserve its utility for the purposes outlined above (section 3.4) whilst ensuring the maintenance of confidentiality. It is recommended that, where possible, a suitable agency be identified, in advance, to store the registry archive for a minimum of 35 years. The agency should undertake to make the database available for the purposes defined by the registry and under the same rules of confidentiality as applied by the registry. Consideration should also be given to the data selected for storage and the method of archiving. Selected paper records might be microfilmed, and selected computer files archived on electronic media. Safe disposal of confidential records not included in an archive deposit should also be planned in advance.

### 7. Summary of conclusions and recommendations

7.1 *Principles of confidentiality and the role of the cancer registry*

- 7.1.1 The purposes for which data collected by the cancer registry are to be used should be clearly defined (section 3.4).
- 7.1.2 Identifiable data may be provided to a clinician for use in the treatment of cancer patients (section 3.4.1).
- 7.1.3 Identifiable data may be transferred to a collaborating registry for the purposes of complete and accurate cancer registration (section 3.4.2).
- 7.1.4 The cancer registry must maintain the same standards of confidentiality as customarily apply to the doctor-patient relationship; this obligation extends indefinitely, even after the death of the patient (section 4.1).
- 7.1.5 It may be necessary to ensure that physicians have legal authority to report cancer, where registration is not compulsory (section 4.3).
- 7.1.6 The scope of confidentiality extends not only to identifiable data about data subjects and data suppliers, but also to other directly or indirectly identifiable data stored in or provided to the registry (sections 4.5 and 4.7).
- 7.1.7 Data on deceased persons should be subject to the same procedures for confidentiality as data on living persons (section 4.6).
- 7.1.8 Guidelines for confidentiality apply not only to data stored on computer, but also to data stored in other forms, such as paper, microfilm, microfiche, etc (section 4.8).

7.2 *Measures for data security*

- 7.2.1 The Director of the registry is responsible for data security (section 5.1).
- 7.2.2 The staff of the registry should sign, as part of their contract of employment, a declaration that they will not release confidential information to unauthorised persons. This declaration should remain in force after cessation of employment (section 5.2).
- 7.2.3 Suitable locks and alarm systems should be installed to conrol access to the registry, and a list of persons authorised to enter the registry should be maintained by the Director (sections 5.3 and 5.4).

7.2.4 The Director should maintain a list of staff members indicating the nature and extent of their access to registry data (section 5.5).

7.2.5 Notices reminding staff of the need to maintain confidentiality should be prominently displayed (section 5.6).

7.2.6 Registry staff are responsible for the confidentiality of all data encountered during active registration (section 5.7).

7.2.7 Cancer registries should consider provision of proof of identity to staff engaged in active registration (section 5.7).

7.2.8 Requests to complement incomplete data should be addressed to a named individual at the source by confidential enquiry (section 5.8).

7.2.9 Identifiable data should not be transmitted by any means (post, telephone, electronic) without explicit authority from the Director or a staff member to whom such authority has been delegated (section 5.9).

7.2.10 Cancer registries should consider use of registered post or courier services for confidential data, as well as separating names from other data for transmission (section 5.9.1).

7.2.11 Precautions should be taken for both physical and electronic security of confidential data sent on magnetic or electronic media (section 5.9.2).

7.2.12 The telephone should be used rarely, if at all, for confidential information, and only under specific constraints, by a staff member specifically authorised to do so (section 5.10).

7.2.13 Use of the computer for confidential data should be controlled by electronic and, if possible, physical measures to enhance the security of the data, including use of a separate room, use of passwords, automatic logging of all attempts to enter the system, and different levels of access to data (section 5.11).

7.2.14 Demonstrations of the computer system should be done with separate and fictitious or anonymised data sets (section 5.11.3).

7.2.15 Special precautions should be taken for the physical security of electronic backup media (section 5.11.4).

7.2.16 Consideration should be given to obtaining expert advice on security against unauthorised remote electronic access, if it is not possible to use isolated data processing systems (section 5.12).

7.2.17 Measures should be taken to ensure the physical security of confidential records held on paper, microfilm, microfiche, etc (section 5.13).

7.2.18 A policy should be developed for safe disposal of confidential waste (section 5.14).

7.2.19 Security procedures should be reviewed at suitable intervals, and consideration should be given to obtaining specialist advice (section 5.5).

7.3 *Release of registry data*

7.3.1 Release of cancer registry data for clinical purposes, for research and for health care planning is central to the utility of the registry, and the registry should develop procedures for data release which ensure maintenance of confidentiality (sections 3.4 and 6).

7.3.2 The Director of the registry is responsible for deciding if requests for data meets the registry's guidelines on confidentiality (section 6.1).

7.3.3 Identifiable information about data subjects or data suppliers should not be released for purposes other than those previously specified by the registry, unless all parties concerned provide written consent for such release (section 6.2).

7.3.4 Physicians should be given access to data needed for management of their patients (section 6.3).

7.3.5 Requests for data to be used for research should include a suitably detailed justification of any need for identifiable data (section 6.4).

7.3.6 Measures should be taken to avoid the possibility that individuals might be identifiable from tables containing cells with very few entries (section 6.5).

7.3.7 Data should not normally be provided to individuals about themselves, unless required by law (section 6.6).

7.3.8 For multi-registry or international studies, identifiable data should not normally be transmitted to other registries or countries (section 6.7).

7.3.9 Enquiries from the press should be referred to the Director of the registry or to a staff member nominated for this purpose (section 6.8).

7.3.10 The Director of the registry should obtain evidence that researchers using registry data will adhere to the registry's guidelines on confidentiality of the data (section 6.9).

7.3.11 It is recommended that registries provide a document describing their procedures and criteria for release of data (especially identifiable data) to researchers who request access to the data (section 6.10).

7.3.12 It is recommended that advance plans should be made for the possible cessation of registry activity, in order to maintain the subsequent utility of the database whilst safeguarding the confidentiality of its data (section 6.11).

### References

MEDICAL RESEARCH COUNCIL. (1985). Responsibility in the use of personal medical information for research: principles and guide to practice. *Br. Med. J.*, **290**, 1120–1124.

PERCY, C., VAN HOLTEN, V. & MUIR, C.S. (eds). (1990). *International Classification of Diseases for Oncology*, Second edition. WHO: Geneva.

WATERHOUSE, J.A.H. (1978). Preface. In MacLennan, R., Muir, C.S., Steinitz, R. & Winkler, A. (eds). *Cancer Registration and Its Techniques* (IARC Scientific Publications No.21). IARC: Lyon, p.ix.